# Appendicitis Perforated by Endometriosis in a 50-Year-Old Woman: A Case Report

**DOI:** 10.7759/cureus.85668

**Published:** 2025-06-09

**Authors:** Omar Alsayed, Joanna E Jayakumar, Osama Alzoabi, Yousif Alabboudi

**Affiliations:** 1 Department of General Surgery, Dubai Academic Health Corporation, Dubai, ARE; 2 Department of Surgery, Mohammed Bin Rashid University of Medicine and Health Sciences, Dubai, ARE

**Keywords:** appendiceal endometriosis, appendicitis, endometriosis, laparoscopic appendectomy, perforated appendix

## Abstract

People often experience pain in the lower right side of the abdomen due to appendicitis, which is a frequent issue for both men and women. Every year, numerous appendectomies are performed in the United States. Another condition that affects many women is endometriosis, where tissue similar to the uterine lining appears in other parts of the body, such as the ovaries, fallopian tubes, or abdominal area. In rare situations, it might even be found in the chest. We report a case of a 50-year-old woman presenting with acute right lower quadrant pain, diagnosed with complicated appendicitis. Laparoscopic appendectomy was performed, and histopathology revealed appendiceal endometriosis at the site of perforation. Appendiceal endometriosis remains a rare and often unsuspected cause of appendicitis, posing diagnostic and surgical challenges.

## Introduction

Appendicitis is a leading cause of right lower quadrant pain in the general population, affecting both men (8.6%) and women (6.7%). Doctors in the United States perform approximately 300,000 appendectomies annually [1]. Endometriosis is characterized by the ectopic presence of functional endometrial glands and stroma outside the endometrium and myometrium, often leading to benign proliferative and inflammatory changes in the surrounding tissues. This tissue is commonly found in the ovaries, fallopian tubes, pelvic peritoneum, and uterosacral ligaments. Still, it can also appear in less typical places such as the gastrointestinal tract, urinary tract, soft tissues, and chest [2]. Moreover, one in every 150 women receives treatment for endometriosis, which makes this a relatively common condition among females of reproductive age [3]. However, the occurrence of endometriosis in the appendix leading to perforated appendicitis is a rare phenomenon. Recently, we encountered a case where endometriosis of the appendix led to a perforated appendix. This highlights the need for further research into this intriguing area, exploring the relationship between endometriosis and appendicitis.

## Case presentation

A 50-year-old woman presented to the emergency department with a two-day history of abdominal pain, mainly in the right lower quadrant. The patient described the pain as nonradiating and intermittent. The patient denied any associated symptoms. The patient had no significant medical, surgical, or family history, except that she was allergic to amoxicillin.

During the physical examination, the patient was vitally stable and afebrile. Abdominal examination revealed guarding with tenderness in the right lower quadrant and positive rebound tenderness in McBurney's point, and a positive Rovsing’s sign. Laboratory investigations showed elevated inflammatory markers C-reactive protein (CRP; 212 mg/L), white blood cells (WBCs; 16.9 × 10³/µL), neutrophils (13.2 × 10³/µL), and elevated random blood sugar (259 mg/dL). Urinalysis positive for urine protein, glucose, and ketone.

**Figure 1:**
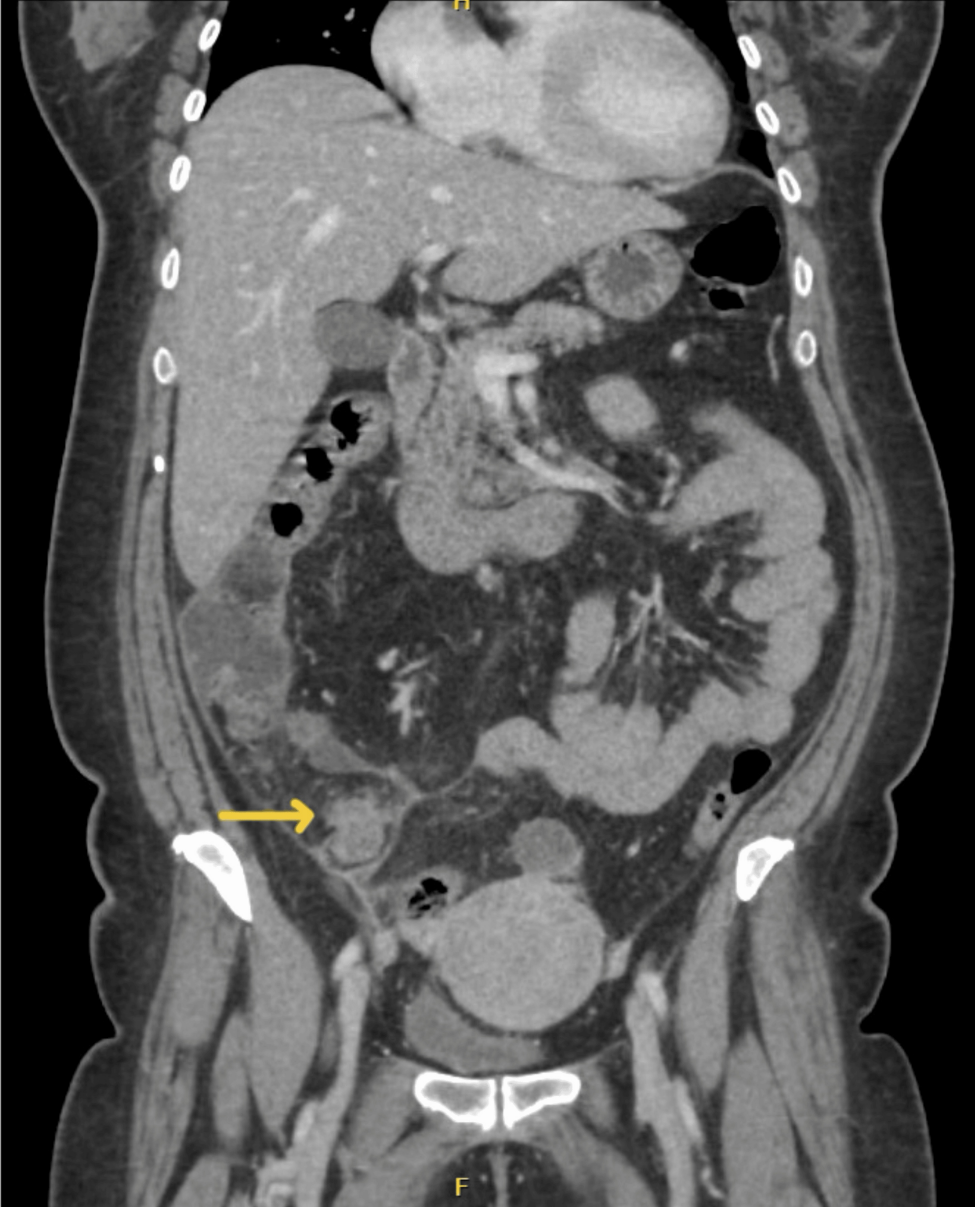
Coronal CT scan of the abdomen and pelvis with IV contrast showing dilated tip of the appendix, around 1.4 cm in diameter, with marked periappendiceal fat stranding and minimal free fluid CT: computed tomography; IV: intravenous

A computed tomography (CT) scan of the abdomen and pelvis with intravenous contrast was done, and findings showed an irregular, distended appendix of 1.4 cm associated with significant periappendiceal inflammatory changes and minimal fluid with thickening of adjacent fascia, and a suggestive diagnosis of acute complicated appendicitis (Figure 1).

The patient was admitted under the General Surgery team and was taken to the operating room immediately, where the patient underwent laparoscopic appendectomy through the standard three-port technique. Intraoperative findings included an inflammatory mass formation in the right iliac fossa adherent to terminal ileal loops and cecum with surrounding fibrin and turbid fluid in the pelvis. The appendix was identified after blunt dissection of surrounding tissue, and the appendix was inflamed and thickened, measuring 6.5 x 1.5 cm. Additionally, a small cyst was noted in the right ovary. Intraoperatively, the gynecologist was contacted regarding the cyst in the right ovary, but it was deemed benign, and no intervention was done regarding the cyst.

**Figure 2:**
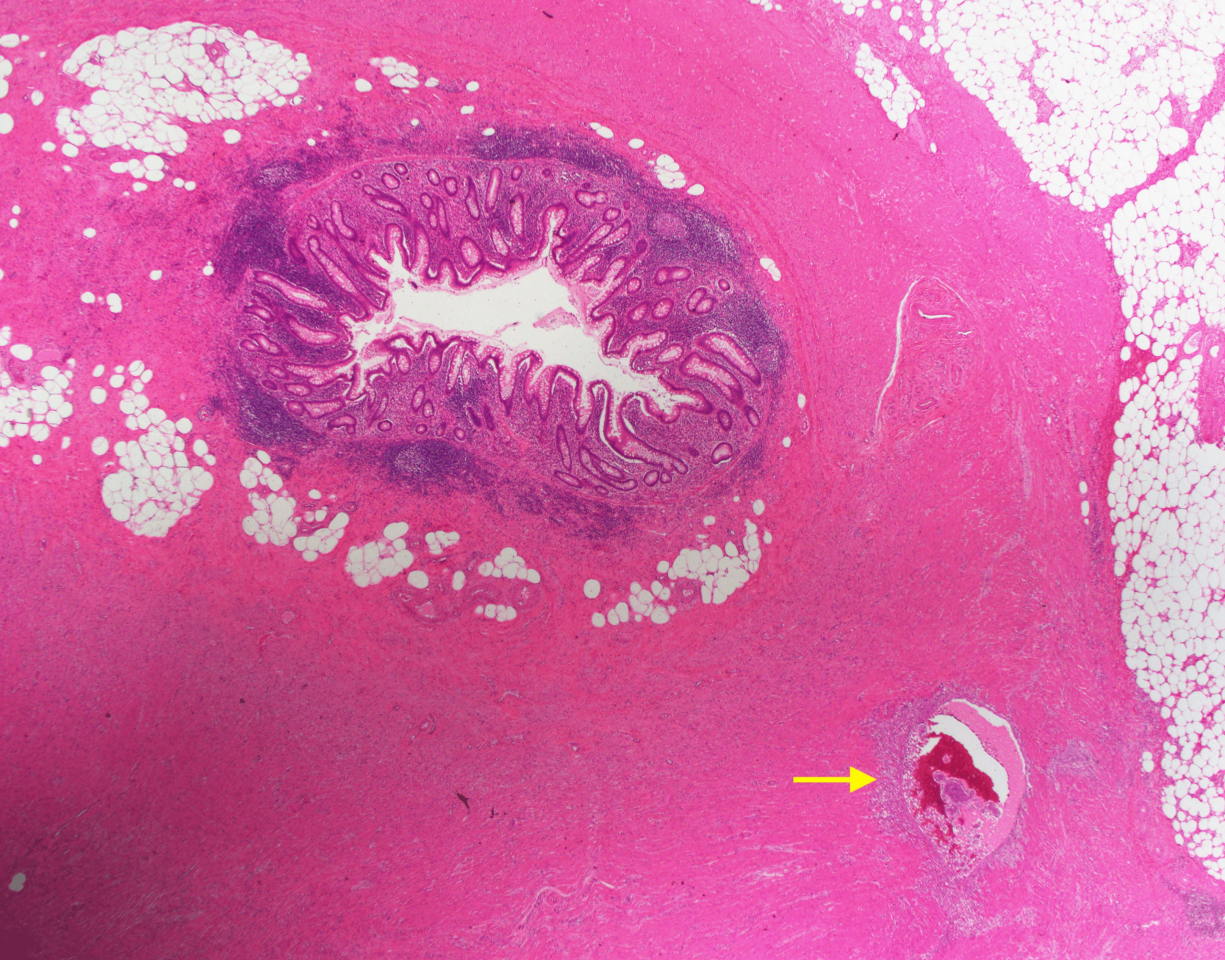
Appendix wall with an intramural hemorrhagic cystic endometriotic foci in the outer muscle layer

**Figure 3:**
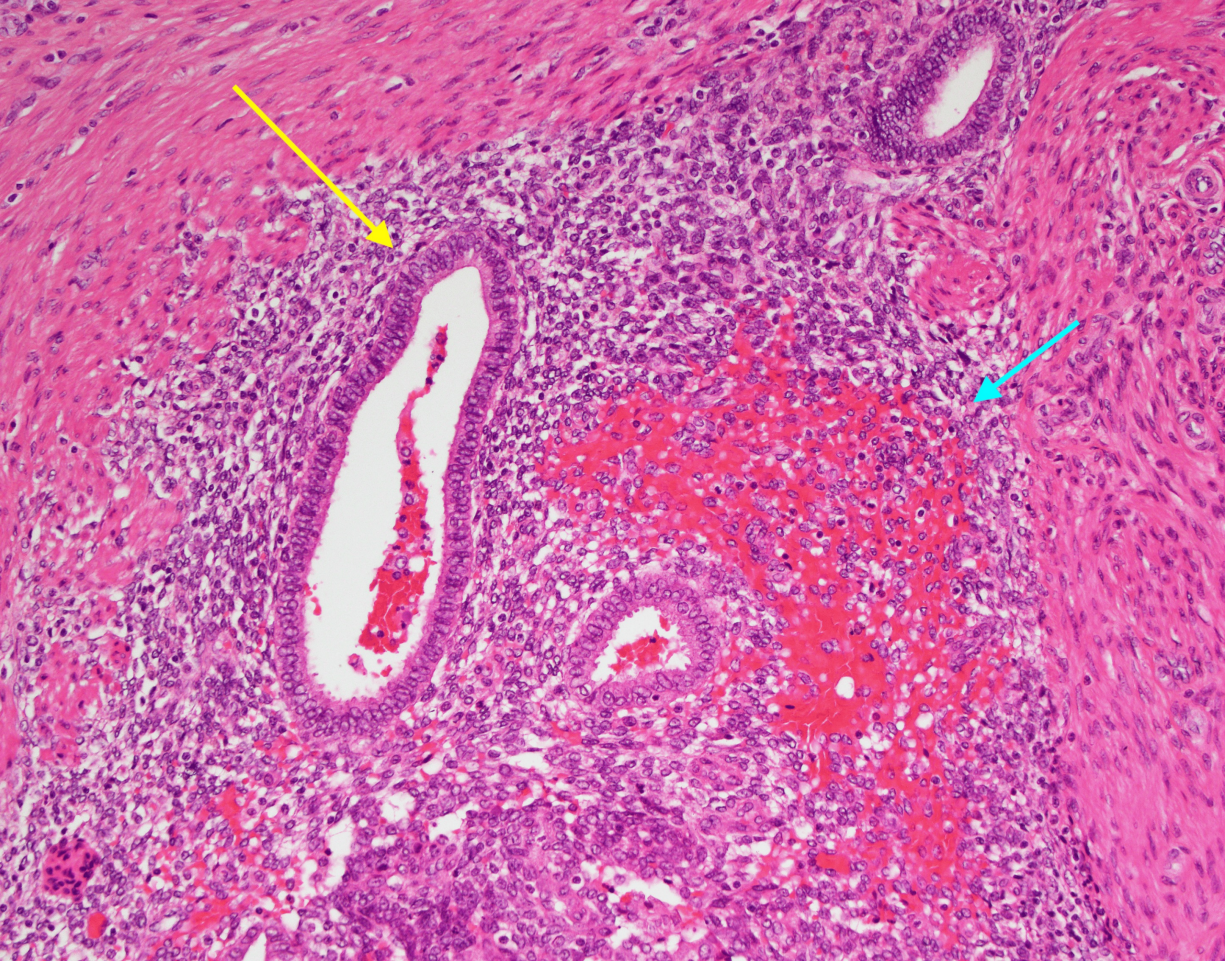
Endometrial glands (yellow arrow) with endometrial stroma and hemorrhage (blue arrow) consistent with endometriosis

Histopathological examination of the removed appendix revealed that the lumen was narrowed and then dilated by a thickened, distorted wall with hemorrhagic spots (Figures 2, 3), with the presence of reactive small periappendiceal lymph nodes. The appendiceal tip showed focal early acute appendicitis. All these findings were suggestive of appendiceal endometriosis.

Postoperatively, the patient was kept on oral antibiotics (cefuroxime and metronidazole) and was discharged on the third day. The patient received a follow-up appointment at the gynecology clinic. However, she was lost to follow-up.

## Discussion

Endometriosis is a chronic, estrogen-dependent gynecological disorder characterized by the ectopic presence of endometrial-like glands and stroma outside the uterine cavity, most commonly on the pelvic peritoneum, ovaries, and rectovaginal septum [4]; 8%-10% of women and adolescents of reproductive age have been diagnosed with endometriosis [5]. The ovaries, pelvic peritoneum, fallopian tubes, and uterosacral ligaments are some of the common locations where endometriosis occurs; however, extrapelvic regions of endometriosis consist of urinary tract, gastrointestinal tract, omentum, mesentery, liver, operation scars, and, rarely, in the kidneys, lungs, central nervous system, skin, and extremities [2].

Among women presenting with symptoms of acute appendicitis, up to 2.8% are ultimately diagnosed with appendiceal endometriosis, compared to a significantly lower incidence of 0.4% in the general population [6]. In 15%-20% of patients with pelvic endometriosis, the intestinal tract is affected, with appendiceal endometriosis observed in 3% of these cases [7,8].

Patients with endometriosis of the appendix can be asymptomatic or they may present with acute or chronic lower abdominal pain [9]. Some of the documented common symptoms include intermittent right lower quadrant pain, bleeding per rectum, intussusception of the cecum, and perforation of the intestine, primarily seen in pregnancy [2,9,10]. Symptomatology can also be classified into four major groups: 1) acute appendicitis presentation, 2) appendicular invagination presentation, 3) atypical presentation including melena, nausea, and colicky abdomen, and 4) asymptomatic presentation.

There are multiple proposed pathogeneses as to how endometriosis may affect the appendix. One possible cause of endometriosis is retrograde menstruation, where endometrial tissue flows backward through the fallopian tubes into the abdominal cavity. This tissue can implant on the peritoneal surface, sometimes affecting the gastrointestinal tract. However, the appendix is not explicitly mentioned as a common site affected by this process [11]. On the other hand, endometriosis in the extragenital regions is not defined yet. However, direct transplantation and dissemination through the oviduct have been proposed as theories for extragenital endometriosis [12]. Furthermore, the ectopic presence of endometrium-like tissue is present in various parts of the musculature, including subserosal and muscularis propria, corroborating the ectopic transplantation theory [12]. In contrast, other studies have identified that endometriosis of the appendix has no association with ovarian pathologies, which counters the theory of ectopic transplantation through the oviduct [13]. Acute appendicitis comes into play when endometriosis in the appendix and menstrual bleeding in ectopic tissue coincide [14]. The appendix becomes inflamed due to partial or complete occlusion of the lumen, primarily caused by endometrioma [15].

The diagnosis of appendiceal endometriosis is challenging. Blood results often show elevated CRP, elevated WBC counts, and the presence of polymorphonuclear leukocytes in acute appendicitis presentation. Abdominal CT scan can be a helpful modality in reaching a definitive diagnosis. The diagnosis of endometriosis on CT secondary to appendicitis illustrates a distended, nonopacified appendix without the presence of inflammation and is not easily distinguished from acute appendicitis [15]. A precise diagnosis is only affirmed after a postoperative histopathological review of the specimen [16].

Routinely, the treatment plan for appendiceal endometriosis is a combined approach, which includes surgery and hormone therapy, and is usually decided on a patient-to-patient basis. Accordingly, the resection should be strategized. The end goal for patients with this presentation consists of appendectomy for appendicitis and laparoscopic surgery for the management of endometriosis, whenever indicated [16]. Appendectomy is a requirement for secure treatment of endometriosis of the appendix [17]. Routine appendectomy in female patients with unexplained chronic pelvic pain was noted to show some improvement in the patients' symptoms. However, it is not yet fully understood [18]. Overall, it helps in determining whether the causal factor was acute appendicitis or worsening symptoms of endometriosis. Postoperatively, an overview gynecological assessment should be performed on the patient to evaluate the extent of endometriosis with regular follow-up for the management of endometriosis secondary to appendicitis [15].

## Conclusions

In conclusion, the case of appendix perforation complicated by endometriosis underscores the complexity and diagnostic challenges associated with both conditions. While appendicitis is a well-known cause of abdominal pain, the rare occurrence of appendiceal involvement in endometriosis highlights the importance of considering atypical etiologies in cases of diagnostic uncertainty, especially in patients with a history of pelvic pain or endometriosis. The management of such cases requires a multidisciplinary approach, with careful consideration of surgical intervention and histopathological examination to confirm the diagnosis. Further research into the relationship between endometriosis and appendicitis is warranted to better understand the underlying mechanisms and optimize treatment strategies for affected individuals.
